# Endoscopic screening for identification of signet ring cell gastric cancer foci in carriers of germline pathogenic variants in *CDH1*

**DOI:** 10.1007/s10689-024-00421-z

**Published:** 2024-09-11

**Authors:** Lady Katherine Mejia Perez, Margaret O’Malley, Arjun Chatterjee, Ruishen Lyu, Qijun Yang, Michael W. Cruise, Lisa LaGuardia, David Liska, Carole Macaron, R. Matthew Walsh, Carol A. Burke

**Affiliations:** 1 Department of Gastroenterology, Hepatology and Nutrition Digestive Disease and Surgical Institute, Cleveland Clinic, Desk A30, 9500 Euclid Avenue, Cleveland, OH 44195 USA; 2 Department of Colorectal Surgery, Cleveland Clinic, Cleveland, OH USA; 3 Sanford R. Weiss MD Center for Hereditary Colorectal Neoplasia, Cleveland Clinic, Cleveland, OH USA; 4 Department of Internal Medicine, Cleveland Clinic, Cleveland, OH USA; 5 Department of Quantitative Health Sciences, Cleveland Clinic, Cleveland, OH USA; 6 Department of Pathology and Lab Medicine, Cleveland Clinic, Cleveland, OH USA; 7 Department of General Surgery, Cleveland Clinic, Cleveland, OH USA

**Keywords:** Hereditary diffuse gastric cancer, Endoscopy, Surveillance, Gastrectomy, *CDH1*

## Abstract

To determine the preoperative detection of signet ring cancer cells (SRC) on upper endoscopy (EGD) in patients with *CDH1* pathogenic variant (PV) undergoing gastrectomy. To evaluate the development of advanced diffuse gastric cancer (DGC) in patients choosing surveillance. Guidelines recommend prophylactic total gastrectomy (pTG) in *CDH1* PV carriers with family history of DGC between 18 and 40 years. Annual EGD with biopsies according to established protocols is recommended in carriers with no SRC and no family history of DGC, with consideration of pTG. Retrospective analysis of asymptomatic patients with *CDH1* PVs with ≥ 1 surveillance EGD. Outcomes included pre-operative EGD detection of SRC, surgical stage, and progression to advanced DGC in those electing surveillance with EGD. 48 patients with *CDH1* PVs who had ≥ 1 EGD were included. 24/ 48 (50%) underwent gastrectomy, including pTG in 7 patients. SRCC were detected on gastrectomy specimen in 21/24 (87.5%). SRCs were identified by EGD in 17/21 patients who had SRCC on gastrectomy specimens (sensitivity 81%, 17/21). All cancers were stage pT1a. The remaining 17 patients (50% with a family history of gastric cancer) continue in annual EGD surveillance with a median follow-up of 34.6 months. No SRCC or advanced DGC have been diagnosed. No *CDH1* PV carriers without SRCC on random biopsies followed in an endoscopic program developed advanced DGC over a median follow up of 3 years. In the short term, EGD surveillance might be a safe alternative to immediate pTG in experienced hands in referral centers.

## Background

*CDH1* pathogenic variants (PVs) are responsible for 40% of cases of hereditary diffuse gastric cancer (HDGC), which is an autosomal dominant syndrome with variable penetrance. The reported cumulative risk of DGC by age 80 years in carriers of *CDH1* PVs is reported to be 33 to 42%, and more recent data suggest that the risk is as low as 7–10% [1–3]. That data is discrepant with prophylactic gastrectomy (pTG) specimens demonstrating up to 97% of asymptomatic PV carriers have early-stage DGC regardless of preoperative detection of SRC [4–12]. Updated guidelines for the management of patients with *CDH1* PVs recommend prophylactic total gastrectomy (pTG) in early adulthood for *CDH1* PV carriers with a family history of DGC regardless of the presence of SRC on endoscopic biopsy while total gastrectomy (TG) is recommended in those with SRC detected on EGD [13]. Upper endoscopy (EGD) surveillance is recommended annually in patients without SRC or a family history of gastric cancer who choose not to undergo surgery. EGD using a specific protocol, such as the IGCLC guideline protocol should include targeted biopsies of pale patches, and mucosal abnormalities and a minimum of 30 random biopsies of six different anatomical areas in the stomach [13]. While SRC DGC is found in 83–97% of pTG specimens, published literature reports 15–61% of patients have SRC DGC detected on preoperative biopsies, even in expert centers [Table 1] [4–12]. As more patients without SRC are opting for endoscopic surveillance and the indolent nature of asymptomatic early stage (pT1a) SRC DGC is being understood, the recommendation of need for and timing of gastrectomy requires careful evaluation of the patient family history, health status, and endoscopic findings. Gastrectomy with Roux-en-Y esophagojejunostomy has a high rate of complications (8–43%) and a great impact on health-related quality of life [14, 15]. Hence, identification of those patients at higher risk of presence of SRC and progressive to advanced stage HDGC is of paramount importance. Likewise, the role of EGD to maximize preoperative detection of SRC DGC and identify endoscopic features suggestive of more advanced stage SRC DGC are needed. Our primary aim was to evaluate the yield of our endoscopic surveillance program for the preoperative identification of SRC DGC in patients undergoing gastrectomy. Our secondary aims were to evaluate factors associated with DGC and outcomes of patients without SRC DGC who opted for surveillance endoscopy.

**Table 1:** Available literature of outcomes of endoscopic surveillance and gastrectomy in patients with *CDH1* PV

Author, year	Number of *CDH1* PV carriers with ≥ 1 EGD; presence or absence of reported symptoms in cohort	Follow-up time from 1st EGD to last observation*, months	Gastrectomy SRCC detection, *n* (%)	Number of patients with EGD detected SRCC without gastrectomy followed with EGD surveillance; follow up from time when SRCC were found to last observation*, months; outcome	Number of patients without EGD detected SRCC followed in EGD surveillance; follow up, months; outcome
Mejia Perez, 2024	48, asymptomatic	34.6	21/24 (88%)	0; all had TG	24; 34.6 months; no DGC detected
Chen et al. [8]	13, asymptomatic	All had gastrectomy after 1st EGD	12/13 (92%)	0; all had TG	0; all had TG
Mi et al. [9]	54, asymptomatic	12	26/28 (93%)	11; f/u not provided for all: 1 progressed to T4aN1M0 DGC after 3.5 years. 10 with progression to advanced DGC	21; 8 months; no DGC detected
Jacobs et al. [10]	20, asymptomatic	Not provided	9/16 (83%)	2; No follow up provided	Not provided
Van Dieren et al. [11]	42, asymptomatic	12	26/30 (87%)	2; One no progression to advanced DGC at 5 years. One no follow-up surveillance done	10; 19.2 months; No DGC detected
Vos et al. [4]	101, asymptomatic	N/A Surgery in all	88/101 (87%)	0; all had TG	0; all had gastrectomy
Friedman et al. [5]	48 symptoms unknown	43	31/32 (97%)	0; all had TG	13; 43 months; one diagnosed with stage II (T3N0M0) DGC after 9.4 years of surveillance. No DGC detected in others.
Laszkowska et al. [6]	98, symptoms unknown	17.9	50/58 (86%)	0; all had TG	17/32, 17.9 months; no DGC detected
Lee et al. [7]	145**, symptoms unknown	51	31/36 (86%)	13; 9–36 months; 6 developed visible lesions^ξ^ and went for TG	Not provided
Asif et al. [12]	270, asymptomatic	31.1	95/98 (97%)	58; f/u time not provided; no progression to advanced DGC	120; f/u time not provided; 2 developed pT3N0M0 DGG on their initial and third EGD

Abbreviations PV = pathogenic variant, TG = total gastrectomy, HDGC = hereditary diffuse gastric cancer

* = last EGD or gastrectomy, ** included 92 *CDH1* PV carriers and 50 patients with HDGC and negative *CDH1* PV carriers; ^ξ^ = visible lesions included pale areas, polyps, erosions, ulcers, nodular lesions, or regions with irregular pits or vasculature

## Methods

### Study design

Consecutive asymptomatic patients from 13 to 90 years old with *CDH1* PVs referred to the Sanford R. Weiss M.D Center for hereditary colorectal neoplasia were retrospectively ascertained from the IRB-approved Jagelman Inherited Colorectal Cancer Registries. Those who underwent at least one EGD from 2007 to 2022 for surveillance were included in this study.

Genotype was confirmed by genetic test reports. Information on demographic characteristics, personal and family history, endoscopic and histological findings, surgical histological findings, and long-term follow-up were collected retrospectively. All patients with a *CDH1* PVs who were clinically fit for surgery, were counseled by a gastroenterologist and foregut surgeon with expertise in the surgical management of hereditary cancer risk in *CDH1*. Those who declined gastrectomy were offered annual EGD surveillance.

### Definitions and outcomes

EGD was performed with high-resolution white-light endoscopes [Olympus GIF HQ 190] to examine all anatomic segments of the stomach. The presence and location of pale areas, nodules, erosions, or polyps was recorded. Targeted biopsies of pale patches were done. In the first few years of surveillance a minimum of 33 random biopsies from five areas of the stomach were obtained according to the IGCLC guideline protocol and evolved to include 77 random 4 quadrant biopsies from 11 areas of the stomach including the cardia, pre-pylorus, antrum (distal, proximal), transition zone, body (proximal, mid, distal), fundus (proximal, mid, distal). Radial jaw, 2.8-mm cold biopsy forceps with needle were used [Boston Scientific]. Double bites were obtained of normal appearing mucosa with each pass of the cold biopsy forceps.

The biopsy specimens were placed in formalin-fixative and stained with hematoxylin and eosin for assessment of SRC by an expert GI pathologist. Multiple sections from the specimen were assessed (typically 4 or 5) and 2 hematoxylin and eosin-stained slides were obtained per case, for a total of 8 to 10 initial sections. The slides were assessed for SRC. If findings were suspicious but not diagnostic of SRC, additional H&E slides (2 or 3) were performed. Gastrectomy specimens were reviewed by an expert GI pathologist based on clinical practice guidelines [13]. Any gross lesions or prior biopsy sites were identified first. If any of these were present, those were submitted for review. If not, the entire stomach was submitted in a stepwise fashion, starting with a set of 10–20 blocks of the fundus, then body, cardia, and antrum until tumor was identified. The margins were submitted entirely.

### Statistical analysis

Data are described using means and standard deviations for normally distributed continuous variables, medians, and interquartile ranges (IQR) for non-normally distributed continuous variables, and frequency (percentage) for categorical variables. A Kaplan-Meier plot was constructed to evaluate the time of endoscopic diagnosis of SRC since the first endoscopy until the last date of follow-up. Patients who underwent gastrectomy, who were lost to follow-up, or in whom the last follow-up had shown no SRC were censored at the date of the last endoscopy. Traditional univariate logistic regression and univariate logistic regression using mixed effect model account for multiple observations of each patient were used to determine the relationship between SRC outcome and predictors of SRC. Predictors included in the analysis were number of random biopsies, endoscopist, and family history of DGC. P-values were provided by likelihood ratio test. All data was collected and managed using RedCap (Research Electronic Data Capture) hosted at the Cleveland Clinic. Analyses were performed using R software. A significance level of 0.05 was assumed for all tests.

## Results

### Patient and procedure characteristics

**Fig. 1:**
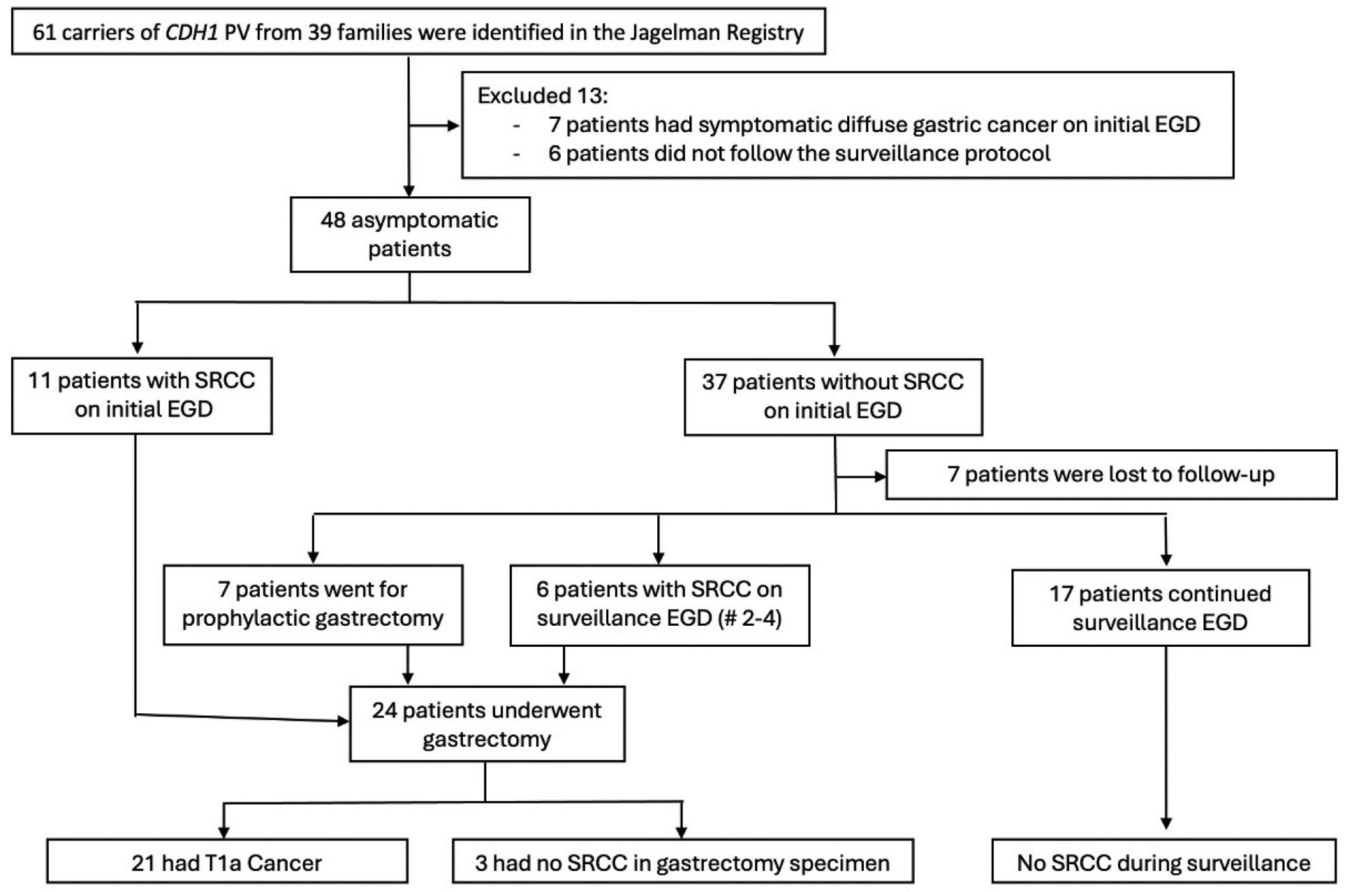
Flow diagram of patients included in the study

Sixty-one carriers of *CDH1* PVs from 39 families were identified [Fig. 1]. Thirteen patients were excluded who presented with symptomatic cancer or did not undergo surveillance EGD at our center. Forty-eight patients underwent 1 to 10 EGDs for a total of 113 examinations. The median age at first EGD was 46.7 years [IQR 32.2, 57.75] [Table 2]. Most patients were female [*n* = 33, 68.7%], white [*n* = 37, 77.0%] and reported a family history of gastric cancer [*n* = 31 (65%)] with histology confirmed to be DCG in 16 (52%) and unknown pathology in 15 (48%).

**Table 2:** Demographic characteristics

Characteristic	*N* = 48	Presence of SRC during EGD surveillance	
		Yes, *N* = 17	No, *N* = 31
Female gender, n (%)	33 (68.7%)	9	24
Age at the time of *CDH1* testing, years, median [IQR]	45.5 [33, 57.5]	43.8 [32.1–56.3]	46.2 [36.6–57.7]
Age at time of 1st EGD, years, median [IQR]	46.7 [32.2, 57.75]	43.9 [30.6–57.3]	45.8 [33.4–57.8]
Age at time of last EGD, years, median [IQR]	47.6 [33.6, 58.8]	44.9 [35.0-53.2]	46.5 [31.1–62.2]
Ethnicity, n (%)			
White	37 (77.0%)	13	24
Black	4 (8.3%)	1	3
Asian	4 (8.3%)	3	1
Other	3 (6.3)	0	3
Personal history of cleft palate, n (%)	1 (2%)	0	1
Personal history of breast cancer, n (%)			
Lobular	9 (18.7%)	4	5
Ductal	2 (4.2%)	1	1
Ductal and lobular	2 (4.2%)	0	2
Family history of gastric cancer*, n (%)			
First degree relative	22 (45.8%)	11	11
Second degree relative	21 (43.7%)	12	9
No family history	17 (35.4%)	3	14

Abbreviations: EGD = esophagogastroduodenoscopy; IQR = interquartile range

* A patient could have both first- and second-degree relatives

The median time from *CDH1* genetic test results to first EGD was 4 months [IQR 1.6, 9.6]. One endoscopist did 84% of the 113 EGDs [Table 3]. Fourteen endoscopists performed the remaining 18 EGDs. The median procedural time was 29 min [IQR 24, 35]. There were no complications during or following any of the procedures. Thirty-six pale areas were described amongst all procedures. In 4 of the pale areas, SRC were found: 2 in the antrum, one in the gastric transition zone (T-zone), and one in the fundus. 43 polyps, 16 erosions, and six ulcers were found. None of them harbored SRC. The median number of random biopsies taken was 77 [IQR 40,77].

**Table 3:** Endoscopic characteristics

Characteristic	
Number of EGDs per patient, n (%)	*N* = 48
1	23 (47.9%)
2	11 (22.9%)
≥ 3	14 (29.1%)
#EGDs per endoscopist, n (%)	*N* = 113
Endoscopist 1	95 (84%)
Endoscopist 2	3 (3%)
Endoscopist 3 and 4	2 (2%)
Endoscopist 5 to 15	1 (9%)
Endoscopic complications	0
Procedure time, median minutes [IQR]	29 [24, 35]
EGD when SRCC were detected, n	*N* = 17
First	11 (64.7%)
Second	2 (11.7%)
Third	3 (17.6%)
Fourth	1 (5.8%)
≥ Fifth	0

Abbreviations EGD = esophagogastroduodenoscopy; SRCC = signet ring cells

### Detection of SRC on preoperative EGD and gastrectomy specimens

Eleven of 48 patients (23%) had SRC on the initial endoscopy. All went on to have TG. Of the remaining 37 who did not have SRC foci found on the first EGD, 6 opted for pTG, 24 continued in EGD surveillance, and there was no data available after the first EGD in 7. Of those 24 patients who continued surveillance, two had SRC found on the second EGD, three on their third EGD, and one on their fourth EGD. All 6 of these patients underwent TG and one patient without SRC opted for pTG after their second surveillance EGD. No SRC were found in the remaining 17 patients on their fifth to tenth EGD. In 80 EGDs ≥ 77 biopsies were taken. In 33, < 77 biopsies were taken.

In total 24 of 48 (50%) patients underwent gastrectomy. Three of the seven patients who had pTG did not have SRC on surgical pathology of the gastrectomy specimen. SRC were detected on gastrectomy specimen in 21/24 (87.5%) patients. All patients with SRC detected on preoperative EGD underwent TG and were found to have stage pT1a cancer. The stage of SRC was pT1a in the 4 of seven patients (57.1%) undergoing pTG who had SRC detected in gastrectomy specimen. The other 3 patients had no SRC detected on gastrectomy specimen.

**Fig. 2:**
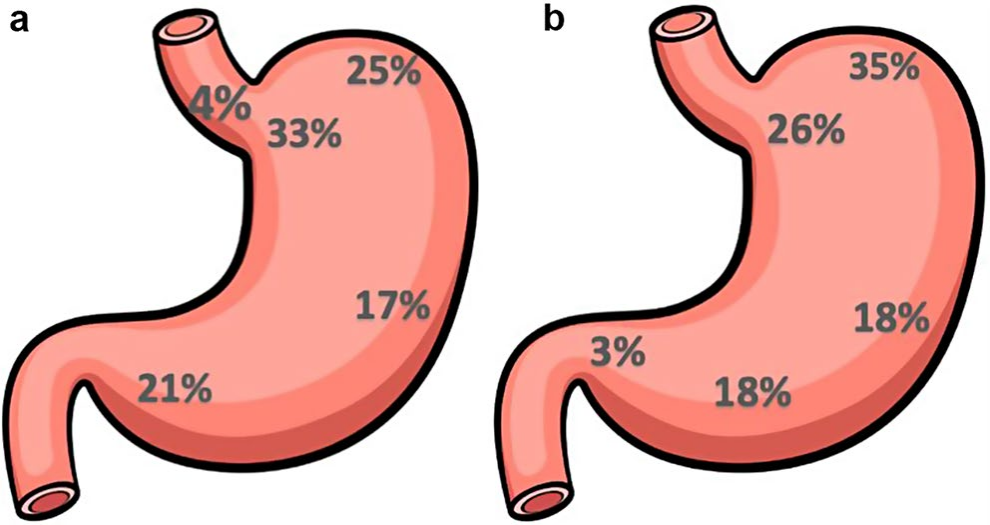
**a** Schematic representation of the location of SRCC detected on EGD. **b** Schematic representation of the location of SRCC detected on surgical specimen

In 6 patients more than one focus of SRC was found on preoperative biopsy. Overall, the SRC foci were in a hiatal hernia sac (4%), fundus (25%), cardia (33%), antrum (21%), and body (17%), [Fig. 2a]. In the gastrectomy specimens, SRC were spread throughout the stomach as well: 26% in the cardia, 35% in the fundus, 18% in the body, 18% in the antrum, and 3% in the pre-pyloric area [Fig. 2b]. SRC was found in 11% of targeted biopsy of pale areas (4/36) including the antrum (2), T-zone (1), and fundus (1).

**Fig. 3:**
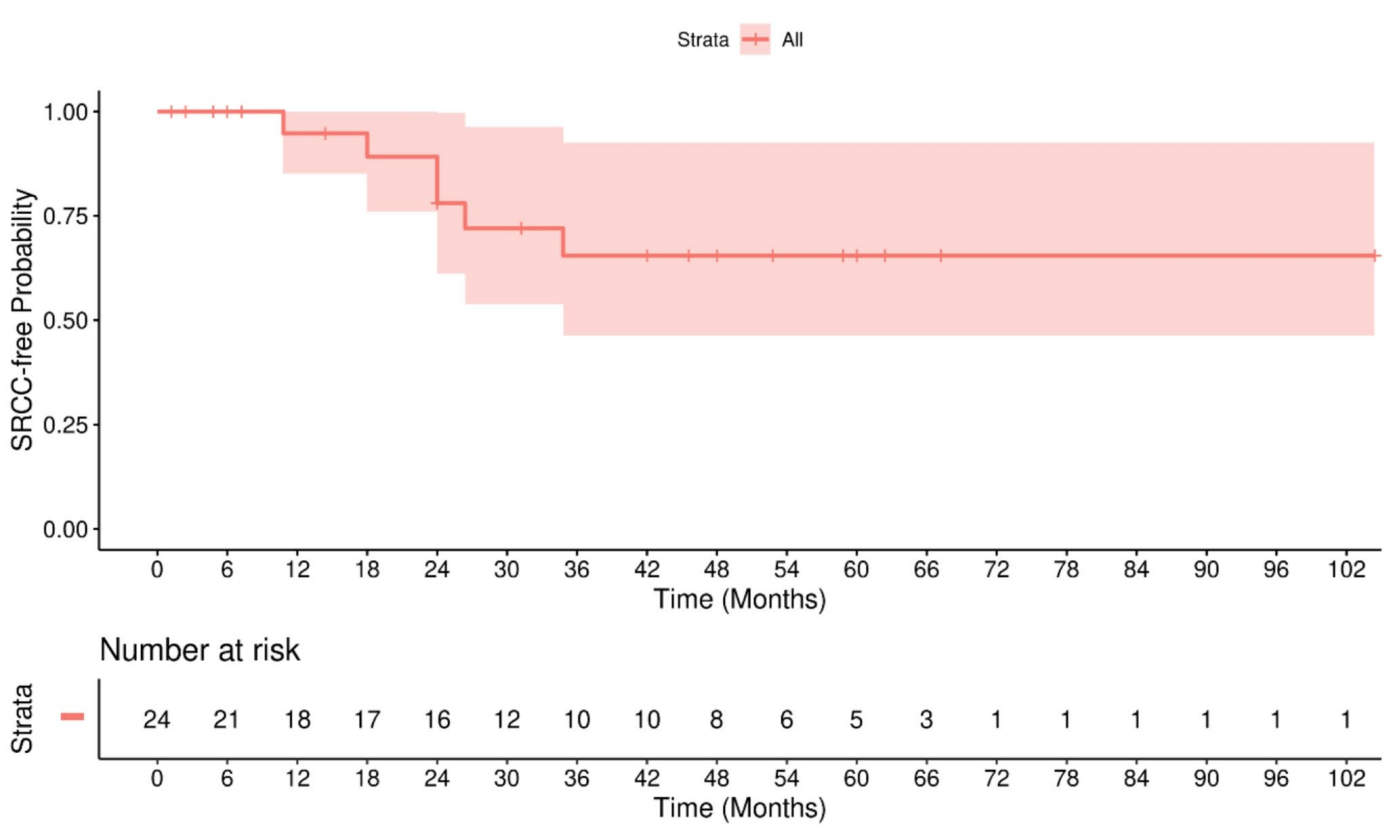
Kaplan Meier curve: Rate of patients with *CDH1* PVs without SRCC detected in the Weiss Center since their first endoscopy. Patients who underwent prophylactic gastrectomy, did not have SCR found during the surveillance time, or were lost to follow-up were censored at the date of the last EGD

**Fig. 4:**
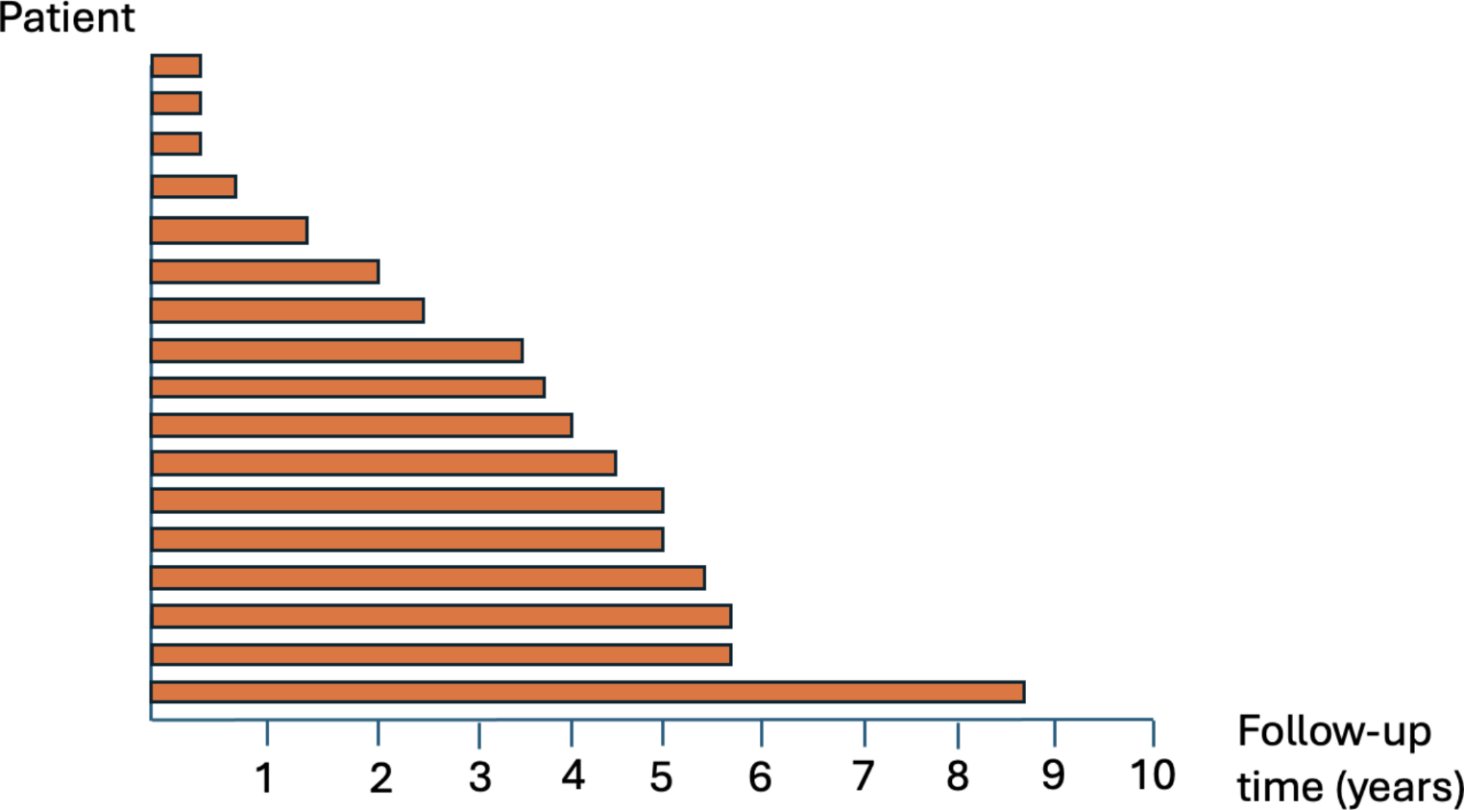
Diagram of the 17 individuals who continue endoscopic surveillance and their respective length of follow up

The 17 patients continued EGD surveillance, continue to have yearly EGDs with a median of 34.6 months of follow up [IQR 12.6,55.8 months] with no SRC detected (Figs. 3 and 4). 9/17 (53%) patients who are still on surveillance EGD had family history of diffuse gastric cancer. 13/17 (76%) patients who had SRC found during surveillance EGD had family history of diffuse gastric cancer. Family History of DGC was statistically significantly associated with the SRC according to the univariable logistic regression analysis [OR = 10.21; Table 4].

**Table 4:** Factors associated with endoscopic detection of signet ring cancer cells

Characteristic	Number of endoscopies	Odds Ratio	95% confidence interval	*p*-value
**Univariate Logistic Regression**				
Number of Biopsies Taken ≥ 77 vs. < 77	113	0.61	0.21, 1.70	0.34
Endoscopist *Main vs. others*	113	1.49	0.38, 4.98	0.54
Family History of DGC *Yes vs. No*	113	5.38	1.63, 24.5	**0.005**
**Univariate Logistic Regression (Mixed Effect)**				
Number of Biopsies Taken *≥ 77 vs. < 77*	113	0.51	0.11, 2.27	0.38
Endoscopist *Main vs. others*	113	1.06	0.15, 7.48	0.95
Family History of DGC *Yes vs. No*	113	10.21	1.27, 82.0	**0.03**

The bold values are statistically significant, as *p* < 0.05

## Discussion

The discrepancy between published literature reporting DGC in 33–42% of patients with *CDH1* PVs by the age of 80 but gastrectomy specimens detecting early stage SRC DGC in up to 97% of carriers needs to be reconciled. Endoscopic diagnosis of SRC is challenging due to the lack of a visible dominant lesion. Prior studies have shown that in order to have a 90% detection rate of SRC, thousands of endoscopic biopsies are required, which is not feasible in clinical practice [16]. Multiple surveillance protocols with different numbers of targeted and random biopsies have been described with limited detection rates from less 12–63% [9, 10, 17–19]. We altered our endoscopic surveillance approach to increase the number of biopsies to enhance the detection of preoperative SRC and describe the sensitivity of our novel protocol.

Increasing the number of random biopsies to 77 and 11 locations in the stomach resulted in a preoperative accuracy of detection of SRC to 81%. This rate is higher than prior studies that have used the IGCLC guideline protocol for preoperative detection of SRC (12–63%).^9,10,17–19^ Our findings that more biopsies are associated with greater SRC detection align with Curtin et al., who recently demonstrated that evolving from the IGCLC guideline protocol to 88 random biopsies increased the preoperative detection of SRC per endoscopy from 15–36% [20]. Importantly, in our center, increasing the number of biopsies was not associated with endoscopic complications and supports the feasibility and safety of our protocol. A variety of other imaging techniques and methods such as endoscopic ultrasound, autofluorescence imaging, confocal endomicroscopy, and chromoendoscopy have been assessed and found to be of no utility for detection of SRC [10, 17, 20–24].

Although SRC were distributed throughout the stomach, they were most found in the proximal stomach, as previously described by authors applying the IGCLC and the Bethesda protocols [17, 20]. This finding suggests that particular attention should be paid to target this area when performing EGD surveillance in this patient population.

Our findings reinforce the concept that more endoscopic biopsies increase the sensitivity of SRC detection but should not discount the utility of targeted biopsies of mucosal abnormalities such as pale patches, nodules, and ulcers [17]. In our study we found that 11% (4/36) of pale patches biopsied on preoperative EGD had SRC foci. Three of the patches that harbored SRC were found on the first endoscopy, and the other one on the second surveillance EGD. The yield of pale patches for SRC detection in the literature varies. Our findings are similar to a recent study reporting 12% of pale areas yielded SRC foci [7]. Other studies have reported SRC detection in pale mucosal areas (specificity 91.7% and sensitivity 57.1%) [6]. There is mixed data about the role of mucosal findings and their association with the presence of SRC and their subsequent development into advanced lesions. Some studies have not found any association between other endoscopic gastric findings, such as polyps, ulcers and erosion, and SRC detection [6, 7], while others have shown that thickened rugal folds, poor distensibility, disturbed vascular patterns, focal ulceration and erosions could identify more advanced SRC DGC [12]. For instance, in a study of 270 *CDH1* PV carries, 120 patients continued endoscopic surveillance and 2 developed clean-based ulcers that harbored SRC on their initial and third EGD. On gastrectomy pT3N0M0 DGC was confirmed [12].

Nearly half of our patients opted to have EGD surveillance instead of pTG when no SRC were detected, while all patients who had preoperative biopsies positive for SRC underwent TG. Most patients (88%) who underwent TG were diagnosed with T1a stage cancer which corroborates the early-stage cancer observed in other asymptomatic cohorts undergoing EGD surveillance [7, 12, 14–16]. None of the patients without SRC in EGD surveillance progressed to advanced gastric cancer during the nearly 3-year follow-up period. It is important to highlight that 50% of these 24 patients in surveillance have a family history of DGC. As 94% of our patients with SRC were detected within the first 3 annual EGDs, the lack of SRC detection on 3 consecutive EGDs in a high detection program may identify a subgroup of patients with low or no disease burden in whom pTG could be deferred. Other centers corroborate our findings that the highest SRC detection occurs on initial endoscopies and no or rare advanced DGC occurs in short term follow up [5, 11]. In our cohort, asymptomatic patients who did not have any SRC found during 3 consecutive annual exams, were not found to have any SRC in subsequent EGDs. More long-term data is needed to establish the safety of lengthening the frequency of exams in those with multiple consecutive normal EGDs.

It is interesting though that in the multivariable analysis, number of SRC was not associated with an increased yield of finding SRC during endoscopic surveillance. The only variable associated with the finding of SRC was family history of *CHD1* PV and HDCG. This might be the result of inheriting certain pathogenic variants associated with higher penetrance of gastric cancer. This finding reinforces the current recommendation of counseling those with family history of HDGC to undergo pTG.

The natural history of asymptomatic SRC detected on EGD is not established. In our cohort, all patients found to have preoperative SRC went for TG, so we could not assess the behavior, natural history nor risk factors for progression from early to advanced SRC cancer. It appears that early stage SRC cancer foci could have an indolent behavior [25]. Emerging data suggests most SRC found in patients on random biopsies represent dormant lesions not necessarily tending to progress into advanced cancer [7, 12]. The ability of endoscopic biopsies to discern between T1a and more advanced lesions is not established. Emerging but limited data of surveillance in patients with SRC suggests the progression to advanced DCG is uncommon. In one series of 22 patients with *CDH1* PV, 1 patient with SRC who did not undergo TG within one year of diagnosis continued at least annual surveillance and developed stage IV DCG 40 months after SRC were first detected [17]. In another study of 42 patients with *CDH1* PV, of 2 patients with SRC who did not have TG, one did not progress to advanced DGC after 7 EGDs over 5 years while the other patient declined surveillance [11]. In a series of 270 *CDH1* PV carriers with a follow up time of 31.1 months, 97% with a family history of gastric cancer, 58 patients were diagnosed with SRC in the absence of a macroscopic abnormality and continued 6 monthly endoscopic surveillance without progression to advanced DGC [16]. Whether endoscopic surveillance could be a widely utilized alternative to TG will require larger studies and longer follow-up demonstrating the absence of progression to advanced cancer. We recommend that surveillance endoscopies for patients with CDH1 PV is done in centers with expert endoscopists who can recognize subtle early SRC lesions. Performance of high-quality endoscopy in CDH1 carriers requires scrutinous visual inspection of meticulously cleansed mucosa. Identification of subtle early SRC lesions is very important and requires significant expertise. This does not discount the importance of random biopsies. A recent study by Lee showed the value of random biopsies in diagnosing advanced disease. Omitting random biopsies would have led to underdiagnosis in their historical cohort of 42%. Additionally, the presence of SRC on random biopsies requires a thorough discussion with the patient of risks and potential treatment strategies. It is paramount to have these discussions and follow-up in centers with expert gastroenterologists and foregut surgeons. The timeframe of progression from early to advanced SRC cancer and the relationship of genotypic factors, histo-morphologic and molecular features, and patient related characteristics, requires better understanding to allow a personalized approach to the need for and timing of both pTG and TG.

This study has several strengths. Our center provides *CDH1* related care to patients from across the U.S. making our patient population potentially generalizable to other expert centers. The follow-up of patients in surveillance without progression to DCG provides additional evidence to the field. While our analysis was retrospective, our patients were consecutive and followed prospectively. We acknowledge bias as we are a referral center with experience in hereditary gastric cancer and one endoscopist performed most of the surveillance EGDs. Our study is small, with short follow-up, and none of our included patients with SRC opted for endoscopic surveillance. However, it is the study reporting the longest follow-up to date. While there were no endoscopic complications from our biopsy protocol, prior biopsy sites on subsequent EGDs appear as superficial mucosal linear scars which in our experience are distinguishable from gastric erosions and classic “pale patches” on both white light and narrow band imaging. The impact of endoscopic biopsy on indolent signet ring cell behavior is not established.

In conclusion, our study shows EGD surveillance in our center by an expert endoscopist with use of the Weiss Center protocol was not associated with endoscopic complications and demonstrated a high preoperative detection of SRC. Over annual surveillance in 24 patients without SRC, including 54% with a family history of DGC, none had progression to DGC over nearly 3 years of surveillance. Over one in ten of our patients who underwent pTG did not have SRC on the gastrectomy specimen. The data our study provides to the accumulating literature allows a more comprehensive management discussion with patients and may reassure patients without SRCs on endoscopic biopsy who are managed in a center with high endoscopic detection of SRC that pTG can be delayed or even avoided. The data to support delaying TG in patients with SRC is limited and the safety of these approaches requires further study.

## Data Availability

No datasets were generated or analysed during the current study.
